# Validation of rapid fecal calprotectin assay using particle enhanced turbidimetric immunoassay for inflammatory bowel disease

**DOI:** 10.1038/s41598-024-51580-z

**Published:** 2024-01-18

**Authors:** Akihiko Oka, Kousaku Kawashima, Kenichi Kishimoto, Satoshi Kotani, Mai Fukunaga, Nobuhiko Fukuba, Yoshiyuki Mishima, Naoki Oshima, Norihisa Ishimura, Muyiwa Awoniyi, Shunji Ishihara

**Affiliations:** 1 Department of Internal Medicine II, Shimane University Faculty of Medicine, 89-1, Izumo, Shimane 693-8501 Japan; 2 Inflammatory Bowel Disease Center, Shimane University Hospital, Izumo, Shimane Japan; 3 Department of Internal Medicine, Matsue Seikyo General Hospital, Matsue, Shimane Japan; 4 Department of Inflammation and Immunity, Digestive Disease and Surgery Institute, Division of Gastroenterology, Hepatology and Nutrition, Cleveland Clinic Lerner Research Institute, Cleveland, OH USA

**Keywords:** Biomarkers, Gastroenterology

## Abstract

Fecal calprotectin (FC) is a promising biomarker for diagnosis and treatment of inflammatory bowel disease, ulcerative colitis (UC), and Crohn’s disease. An enzyme immunoassay (EIA) is widely used for FC detection, though the considerable lag time, up to several days, causes clinical management delay. This study was performed to examine the new rapid kit fCAL-turbo, which is based on a particle-enhanced turbidimetric immunoassay (15 min), by comparing FC values with other EIAs (EliA, PhiCal, Bühlmann) and endoscopic scores. Using 94 samples, fCAL-turbo showed strong significant positive correlations with the other kits (Spearman’s r = 0.9178–0.9886). Of 74 UC patients, 69 underwent an endoscopy and fCAL-turbo reflected endoscopic activity with a moderate correlation with Mayo endoscopic subscore (MES) (r = 0.6945, others r = 0.6682–0.7013). Receiver operating characteristic analyses based on MES 0 versus 1–3 showed a similar efficacy as compared to the other kits (cut-off and area under the curve: 89.70 µg/g and 0.8592, respectively, others 62.35–138.4 µg/g and 0.8280–0.8611, respectively). Furthermore, multiple regression analysis confirmed that fCAL-turbo results significantly contributed to prediction of MES 0 with a higher t-value as compared to the other biomarkers. fCAL-turbo showed strong correlations with the other kits and also demonstrated excellent performance for predicting endoscopic remission of UC.

## Introduction

Inflammatory bowel disease (IBD), represented by ulcerative colitis (UC) and Crohn's disease (CD), is a group of chronic immune-mediated intestinal inflammatory diseases^1–3^. The goal of IBD treatment is to achieve mucosal healing, which reduces hospitalization and need for bowel resection^4–7^. Thus, several endoscopic and histological scoring systems for evaluating mucosal healing have been established and shown to contribute to improvement of prognosis of IBD cases^8,9^. However, frequent use of endoscopy and mucosal biopsy procedures is relatively invasive, especially in children and elderly patients^10^. Therefore, recent research findings have led to development of less-invasive biomarkers for examinations of serum or fecal samples^11^.

Fecal calprotectin (FC) is one of the most promising biomarkers for diagnosis and treatment of IBD^11^. It is primarily derived from granulocytes and intestinal epithelial cells, and has a direct antibacterial effect to regulate inflammatory processes^12,13^. The level of FC reflects migration of granulocytes responding to inflammatory stimuli, thus this biomarker has been used to differentiate intestinal inflammatory disorders, including IBD, from functional intestinal disorders, such as irritable bowel disease, with high levels of sensitivity and specificity noted^11,14,15^. Use of this less-invasive biomarker can help to avoid overlooking of IBD, and also reduce frequent or unnecessary colonoscopy procedures^10^. In addition to screening, FC has been found to be useful for monitoring disease activity and based on strong evidence recommended for clinical practice focused on IBD cases^4,9,16–27^. We and others have also confirmed that FC is correlated with endoscopic remission in a manner superior to other blood biomarkers, such as C-reactive protein (CRP), hemoglobin, platelets, and white blood cell count (WBC)^16–19,21,27^. Moreover, it has been reported that its level shows elevation at two to three months before clinical relapse in IBD patients^28^. Thus, FC is regarded as one of the best biomarkers for prediction of endoscopic remission and clinical relapse in patients with IBD.

Several kits for detection of FC are available. Because of its accuracy and accumulated evidence, enzyme immunoassay (EIA) testing procedures, such as enzyme-linked immunosorbent assay (ELISA), and fluoroenzyme and chemiluminescence immunoassays, are considered to represent the gold standard^11^. EIA testing uses batch analysis and is normally performed by a specialist outside of the treating hospital^29^, thus results can be delayed for up to several days despite the 2.5-h EIA run-time. The present study was conducted to validate fCAL-turbo, a newly introduced rapid automated FC detection kit, in an IBD patient population. An fCAL-turbo assay is conducted as a particle-enhanced turbidimetric immunoassay and can be performed by a general chemical analyzer typically employed by a hospital, with the result available in approximately 15 min^30^. However, its clinical utility for IBD cases has not been fully elucidated. Therefore, we aimed to validate the performance of the fCAL-turbo assay by comparing results with other standard EIA assays in IBD patients. Furthermore, its efficacy for correlations with endoscopic activity^31–33^ and prediction of endoscopic remission in those patients was investigated.

## Results

### Patient demographics

**Figure 1:**
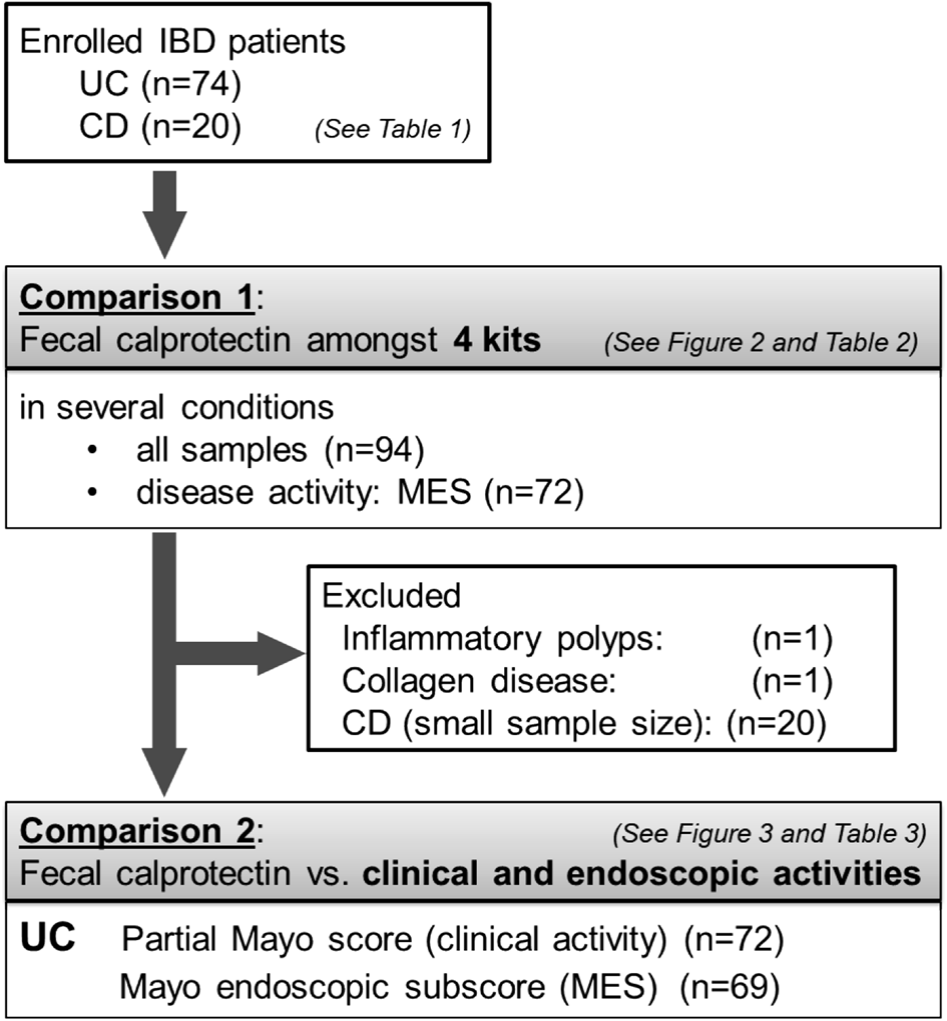
IBD patients included in this study. IBD: inflammatory bowel disease, UC: ulcerative colitis, CD: Crohn’s disease, MES: Mayo endoscopic subscore.

A total of 94 IBD patients (74 UC, 20 CD) were included in this study. Patient profiles are shown in Table 1. Wide range of patients in age (range 20–83 years old), disease activity (78.2% and 75.0% of remission, and 21.8% and 25.0% of active in UC and CD, respectively) and medications (i.e. immunomodulators, steroid, biological drug) were included, allowing more universal validation of FC assays in variety of patients’ conditions. All fecal samples (n = 94) were included in the first comparison analysis (FC values comparison among four kits) (Fig. 1). For the second comparison (FC values vs. clinical and endoscopic activities), results of 22 cases were excluded, as one had multiple inflammatory polyps found by colonoscopy, which can elevate the fecal calprotectin level^34^, and one had collagen disease (polyarteritis nodosa), which can elevate the FC level, which left the remaining sample size of 20 CD patients too small for this comparison analysis. For comparison with endoscopic activities, results from three patients unable to undergo an endoscopy colonoscopy examination for personal reasons were excluded. Finally, a total of 69 UC patients were analyzed using Mayo endoscopic subscore (MES) (Fig. 1).

**Table 1:** Clinical and demographic characteristics of IBD patients in this study.

	UC	CD
Samples, *n*	74	20
Sex (female/male)	25/49	7/13
Age median [IQR]	49.0 [39.8–58.0]	41.5 [36.0–60.3]
Disease activity^a^ (%)		
Remission stage	78.2	75.0
Active stage	21.8	25.0
Duration of disease (median years) [IQR]	6.7 [2.3–11.6]	10.4 [4.4–18.9]
Disease extent^b^ (%)		
Proctitis [E1]	16.2	−
Left-sided-colitis [E2]	31.1	−
Pancolitis [E3]	52.7	−
Disease location^b^ (%)		
Ileum [L1]	−	30.0
Colon [L2]	−	0.0
Ileum + colon [L3]	−	70.0
Upper GI [L4]	−	0.0
Behaviour^b^ (%)		
Non-constricting, non-penetrating [B1]	−	20.0
Stricturing [B2]	−	60.0
Penetrating [B3]	−	10.0
Stricturing and penetrating [B2 + 3]	−	10.0
Medication (%)		
5-aminosalicylate	83.0	88.2
Azathioprine/6-mercaptopurine	39.7	11.8
Anti-TNFα agents	9.4	41.2
Vedolizumab	1.9	0.0
Steroid	11.3	5.9
Tacrolimus	6.8	0.0
Endoscopic activity		
MES = 0/1/2/3	23/19/20/7	−
mSES-CD = 0–2/3–6/7–	−	10/4/6

*IBD* inflammatory bowel disease, *UC* ulcerative colitis, *CD* Crohn’s disease, *IQR* interquartile range, *GI* gastrointestinal. ^a^Assessment of clinical disease activity by using partial Mayo score for UC (remission: 0–2 points, active: 3–9 points) and Crohn's Disease Activity Index (CDAI, remission: 0–150 points, active: > 150 points). ^b^Assessment of disease extent, disease location, and disease behaviour by using the Montreal classification. *TNF* tumor necrosis factor, *MES* Mayo endoscopic subscore, *mSES-CD* modified simple endoscopic score for CD.

### fCAL-turbo kit shows strong positive correlations with other kits

**Figure 2:**
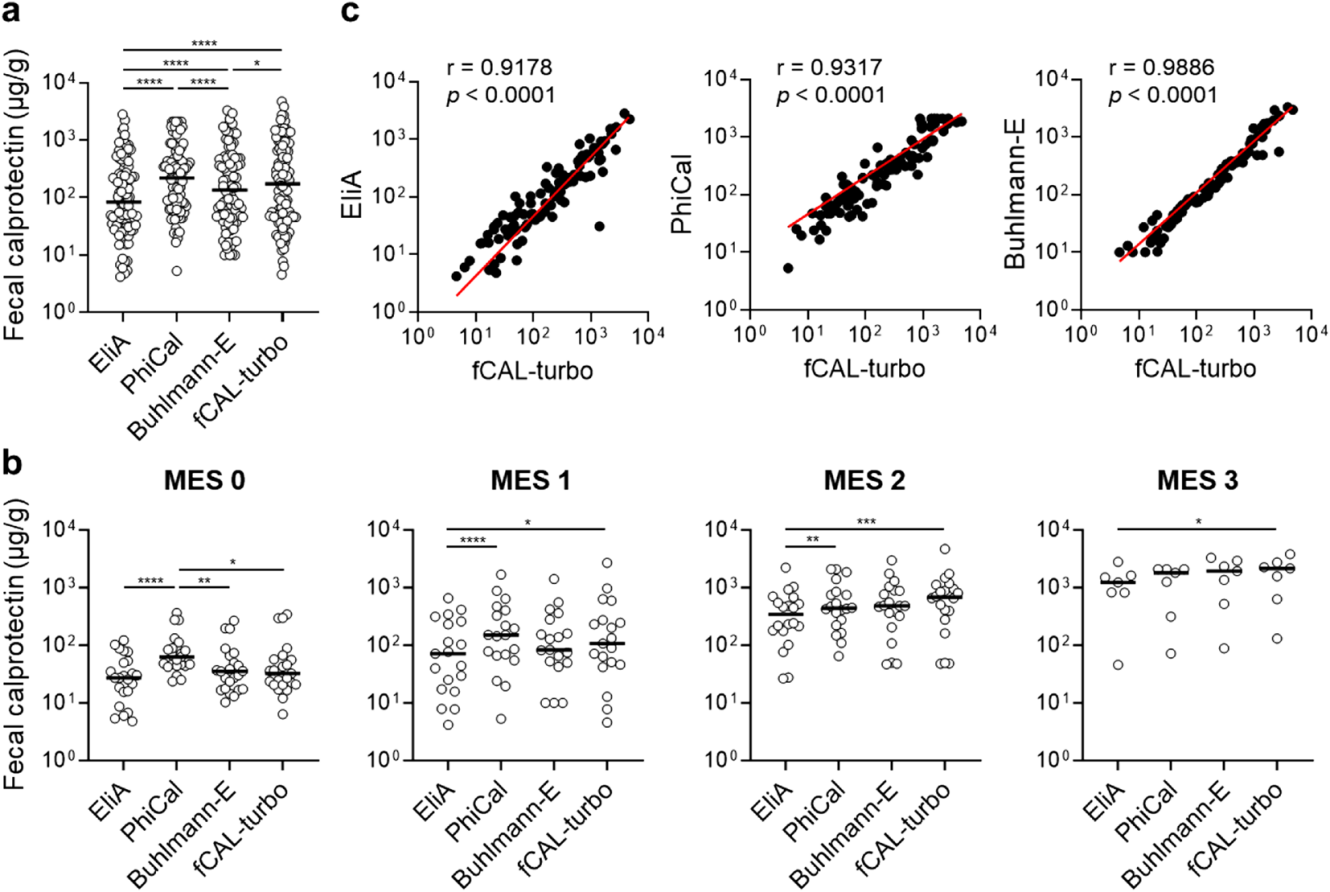
Fecal calprotectin values obtained with examined kits. (**a**, **b**) Fecal calprotectin concentrations determined by use of the EliA, PhiCal, Buhlmann-E, and fCAL-turbo kits are shown for all of the samples (**a**. n = 94) and several disease conditions (**b**. n = 69; MES 0/1/2/3 = 23/19/20/7). Dunn’s multiple comparison test with Friedman’s test was used for the analyses. *p < 0.05, **p < 0.01, ***p < 0.001, ****p < 0.0001. (**c**) Spearman’s rank correlation analysis. Spearman’s correlation coefficient (r) values are shown. MES: Mayo endoscopic subscore.

All four of the examined kits detected calprotectin in each of the fecal samples (n = 94). In the first comparison analysis using all samples, the median FC value varied significantly among the kits [median values: EliA 84.35 [95% confidence interval (CI) 49.80–165.5], PhiCal 220.1 (95% CI 140.6–322.4), BÜHLMANN fCAL^®^ ELISA (hereinafter referred to “Buhlmann-E”) 135.5 (95% CI 77.80–231.2), fCAL-turbo 173.8 (95% CI 89.50–295.4) (Fig. 2a). As for average fold changes, fCAL-turbo showed a 2.0-, 1.2-, and 0.7-fold change of median FC value from EliA, Buhlmann-E, and PhiCal, respectively. Such variations among the kits were consistently observed for all disease conditions (MES 0–3) (Fig. 2b). Interestingly, the fCAL-turbo FC values tended to be lower with lower disease activities (MES 0–1) and higher with increased disease activities (MES 2–3), which suggests a characteristic advantage for use of fCAL-turbo to more specifically detect disease activities. Spearman’s rank correlation analysis demonstrated that fCAL-turbo had a significantly positive correlation with each of the other kits [compared to: EliA (r = 0.9178, 95% CI 0.8775–0.9453), PhiCal (r = 0.9317, 95% CI 0.8978–0.9546), Buhlmann-E (r = 0.9886, 95% CI 0.9827–0.9925)] (Fig. 2c, Table 2).

**Table 2:** Correlation analysis between FC kits in all samples.

Assay X	Assay Y	n	Spearman's rank correlation	
			r (95%CI)	*P value*
fCAL-turbo	EliA	94	0.9178 (0.8775–0.9453)	< 0.0001
fCAL-turbo	PhiCal	94	0.9317 (0.8978–0.9546)	< 0.0001
fCAL-turbo	Buhlmann-E	94	0.9886 (0.9827–0.9925)	< 0.0001
PhiCal	Buhlmann-E	94	0.9265 (0.8901–0.9511)	< 0.0001
PhiCal	EliA	94	0.8970 (0.8472–0.9311)	< 0.0001
Buhlmann-E	EliA	94	0.9281 (0.8925–0.9522)	< 0.0001

*FC* fecal calprotectin, *CI* confidence interval.

### fCAL-turbo kit results reflect clinical and endoscopic activities in UC patients

**Figure 3:**
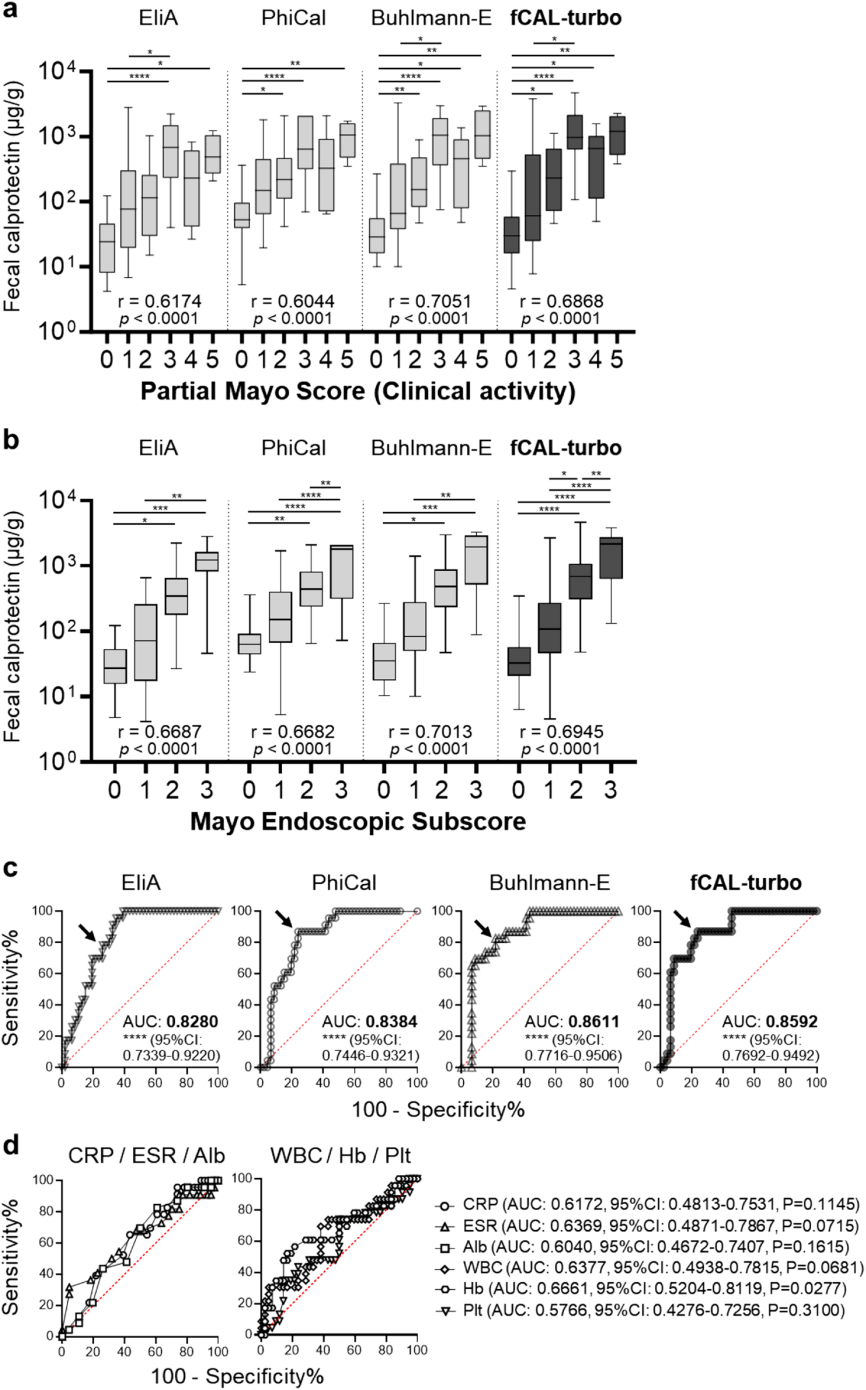
Comparison of fecal calprotectin with clinical and endoscopic activities in ulcerative colitis cases. (**a**) Fecal calprotectin and clinical activity score (partial Mayo score, pMayo). Spearman’s rank correlation analysis was used. Spearman correlation coefficient (r) values are shown (n = 72; pMayo 0/1/2/3/4/5 = 17/19/15/11/6/4). (**b**) Fecal calprotectin and Mayo endoscopic subscores. Spearman’s rank correlation analysis was used. Spearman correlation coefficient (r) values are shown. Kruskal–Wallis multiple comparison testing with ANOVA was performed (n = 69; MES 0/1/2/3 = 23/19/20/7). (**c**, **d**) Receiver operating characteristic curves. Arrows indicate data points with shortest distance from top left corner. Kruskal–Wallis multiple comparison testing with ANOVA was performed for data presented in panels (**a**) and (**b**). *p < 0.05, **p < 0.01, ***p < 0.001, ****p < 0.0001. CRP: C-reactive protein, ESR: erythrocyte sedimentation rate, Alb: albumin, WBC: white blood cell, Hb: hemoglobin, Plt: platelet, AUC: area under the curve, CI: confidence interval.

Results of the second analysis of correlation with UC clinical activity demonstrated that findings obtained with each kit reflected that activity, shown by partial Mayo score (pMayo), with a moderately significant correlation (Fig. 3a, Table 3). Spearman’s rank correlation analysis showed the following results: EliA, r = 0.6174 (95% CI 0.4445–0.7459); PhiCal, r = 0.6044 (0.4277–0.7366); Buhlmann-E, r = 0.7051 (0.5611–0.8077); and fCAL-turbo, r = 0.6868 (0.5363–0.7950). Since mucosal healing is the ideal goal of IBD therapy and endoscopic evaluation is more sensitive than clinical score, correlation analysis was also performed using endoscopic score (MES) (Fig. 3b, Table 4). Each kit reflected endoscopic activity, i.e., MES, with a moderately significant correlation. Spearman’s rank correlation analysis showed the following results: EliA, r = 0.6687 (95% CI: 0.4921–0.7925); PhiCal, r = 0.6682 (0.4913–0.7921); Buhlmann-E, r = 0.7013 (0.5372–0.8142); and fCAL-turbo, r = 0.6945 (0.5277–0.8097). As expected, FC values for endoscopic remission were significantly lower as compared to those for clinical remission (data not shown). Receiver operating characteristic (ROC) curve analysis for differentiation between endoscopic remission (MES = 0) and endoscopic activity (MES = 1–3) showed that the efficacy of the fCAL-turbo kit was similar to that of the other standard kits for the present UC cases (Fig. 3c, Table 5). The optimal cut-off values were 62.35, 138.4, 72.60, and 89.70 µg/g for the EliA (area under the curve, (AUC): 0.8280, 95% CI 0.7339–0.9220, *p* < 0.0001), PhiCal (AUC: 0.8384, 95% CI 0.7446–0.9321, *p* < 0.0001), Buhlmann-E (AUC: 0.8611, 95% CI 0.7716–0.9506, *p* < 0.0001) and fCAL-turbo (AUC: 0.8592, 95% CI 0.7692–0.9492, *p* < 0.0001) kits, respectively. Using the optimum cut-offs, FC values predicted endoscopic remission with a sensitivity of 78.26–86.96%, specificity of 73.91–78.26%, positive prediction value of 59.99–65.51%, negative prediction value of 87.17–92.10%, and accuracy of 75.36–79.71%. The AUC value obtained with the fCAL-turbo kit for predicting endoscopic remission was compared with values obtained with Elia, PhiCal, and Buhlmann-E, and found to be not significantly different (*p* = 0.251, *p* = 0.355, and *p* = 0.780, respectively). These results indicate that fCAL-turbo is a promising FC kit for predicting remission with an efficacy similar to the other FC kits tested. Finally, other clinical biomarkers for IBD, such as CRP, erythrocyte sedimentation rate (ESR), hemoglobin, and others, also useful for predicting remission, were examined, which showed that the AUC obtained with each of each of those was significantly smaller than the fCAL-turbo result (Fig. 3d). Moreover, multiple regression analysis confirmed that fCAL-turbo was the most effective, with a significantly higher t-value for the biomarkers CRP, ESR, hemoglobin, WBC, platelets, and albumin (Table 6).

**Table 3:** Correlation analysis between FC values and pMayo score.

vs. pMayo score	n	Spearman's rank correlation	
Assay		r (95% CI)	*P* value
EliA	72	0.6174 (0.4445–0.7459)	< 0.0001
PhiCal	72	0.6044 (0.4277–0.7366)	< 0.0001
Buhlmann-E	72	0.7051 (0.5611–0.8077)	< 0.0001
fCAL-turbo	72	0.6868 (0.5363–0.7950)	< 0.0001

*FC* fecal calprotectin, *pMayo* partial Mayo, *CI* confidence interval.

**Table 4:** Correlation analysis between FC values and MES.

vs. MES	n	Spearman's rank correlation	
Assay		r (95% CI)	*P* value
EliA	69	0.6687 (0.4921–0.7925)	< 0.0001
PhiCal	69	0.6682 (0.4913–0.7921)	< 0.0001
Buhlmann-E	69	0.7013 (0.5372–0.8142)	< 0.0001
fCAL-turbo	69	0.6945 (0.5277–0.8097)	< 0.0001

*FC* fecal calprotectin, *MES* Mayo endoscopic subscore, *CI* confidence interval.

**Table 5:** Sensitivity, specificity, predictive value, and accuracy of FC kits for predicting endoscopic mucosal healing in UC patients.

Assay	Cut-off (ug/g)	Sensitivity (95%CI)	Specificity (95%CI)	PPV (95%CI)	NPV (95%CI)	Accuracy (95%CI)
EliA	62.35	78.26 (58.10–90.34)	73.91 (59.74–84.40)	59.99 (41.91–74.32)	87.17 (74.03–94.58)	75.36 (59.19–86.38)
PhiCal	138.4	86.96 (67.87–95.46)	76.09 (62.06–86.09)	64.51 (47.21–77.43)	92.10 (79.43–97.43)	79.71 (63.99–89.21)
Buhlmann-E	74.60	82.61 (62.86–93.02)	78.26 (64.43–87.74)	65.51 (46.91–79.13)	90.00 (77.62–96.17)	79.71 (63.90–89.50)
fCAL-turbo	89.70	86.96 (67.87–95.46)	76.09 (62.06–86.09)	64.51 (47.21–77.43)	92.10 (79.43–97.43)	79.71 (63.99–89.21)

*FC* fecal calprotectin, *UC* ulcerative colitis, *CI* confidence interval, *PPV* positive predictive value, *NPV* negative predictive value, *CI* confidence interval.

**Table 6:** Multiple regression analysis of biomarkers for predicting endoscopic remission in UC patients.

Factor	*β*-coefficient (95%CI)	*t* value	*P* value
WBC	− 0.00007 (− 0.00014 to 0.00000)	− 1.984	0.052
Hb	0.10134 (0.02030–0.18238)	2.503	0.015
Plt	0.00804 (− 0.00513 to 0.02123)	1.221	0.227
Alb	− 0.01045 (− 0.38588 to 0.36496)	− 0.055	0.956
CRP	− 0.13058 (− 0.60893 to 0.34775)	− 0.546	0.587
ESR	0.00571 (− 0.00871 to 0.02015)	0.792	0.431
fCAL-turbo	− 0.00018 (− 0.00030 to − 0.00006)	− 3.006	0.004

*UC* ulcerative colitis, *CI* confidence interval, *CI* confidence interval, *WBC* white blood cell, *Hb* hemoglobin, *Plt* platelet, *Alb* albumin, *CRP* C-reactive protein, *ESR* erythrocyte sedimentation rate.

## Discussion

This relatively large comparative study was performed to examine the capabilities of fCAL-turbo, a new rapid FC detection kit. A strong correlation with other well-established EIA kits was noted and the findings indicate that fCAL-turbo accurately reflects endoscopic activity, thus providing useful results for detection of endoscopic remission. Accordingly, it is considered that the fCAL-turbo kit has a clinical efficacy similar to conventional FC kits when used for management of IBD patients. In the updated Selecting Therapeutic Targets in IBD initiative (STRIDE-II), which presents treat-to-target strategies associated with therapeutic goals, reduction of FC to an acceptable range has been proposed as a formal intermediate treatment target^35^. Because of its significant advantage of immediate turnaround time, as well as compatibility and cost performance, use of the fCAL-turbo can enhance the clinical efficacy of treat-to target strategies employed in IBD practice.

To date, three excellent studies have presented comparisons of values obtained with various kits including fCAL-turbo, though did not include endoscopic disease activity^29,30,36^. Oyaert et al.^36^ compared the diagnostic accuracy of six different assays using fecal samples from patients with CD (n = 15), UC (n = 12), gastrointestinal diseases used as a control (n = 52), and rheumatologic disease (n = 26). All six assays including fCAL-turbo demonstrated excellent diagnostic accuracy with similar AUCs. In the study by Noebauer et al.^29^, fecal samples from 95 symptomatic children suffering from chronic diarrhea, abdominal pain, and bloody stool were analyzed using fCAL-turbo, Buhlmann-E, and Quantum Blue, and a good correlation between fCAL-turbo and the other assays was found. Furthermore, Nilsen et al.^30^ compared FC values obtained with the fCAL-turbo and Buhlmann-E kits, and also analyzed variations between those two clinical chemistry analyzers, with a good correlation demonstrated. The present findings support those presented in these previous studies. In addition, we compared FC values and endoscopic disease activity to explore whether the fCAL-turbo assay can provide reliable detection at any disease stage. The findings showed detection of calprotectin at all stages of disease and clearly revealed endoscopic activity in IBD patients.

To the best of our knowledge, this is the first study to use endoscopic score for validation of performance of the fCAL-turbo kit. In UC patients, findings obtained with fCAL-turbo had a strong positive correlation with endoscopic activity (MES). The AUC value for the fCAL-turbo (0.85) for detecting endoscopic remission (MES = 0) was consistent with those of the other assays (0.77–0.86) examined in this study and presented in previous reports, such as PhiCal (cut-off: 180 µg/g; AUC 0.67)^24^, Buhlmann-E (cut-off: 201 µg/g, AUC 0.88) and Quantum Blue (cut-off: 150.5 µg/g; AUC 0.88)^26^, and EliA Calprotectin 2 (cut-off: 146.0 µg/g; AUC 0.777)^25^. Therefore, it is anticipated that the fCAL-turbo kit will be considered to be a reliable tool to monitor UC activity. While data for CD patients are not presented because of the small sample size (n = 20), promising results have shown that fCAL-turbo and other kits, except for EliA, reflect endoscopic activity well, as shown by modified simple endoscopic score for CD (mSES-CD), though the strength of the correlation was found to be moderate, weaker than that in UC patients, which is similar to results presented in previous reports^37,38^. Nevertheless, FC remains a promising biomarker for CD, as we and others have demonstrated its greater accuracy for predicting endoscopic remission in CD patients as compared to serum biomarkers^17,35^. Further comprehensive investigations are required^35^.

As noted in other reports^39,40^, the present findings showed kit-dependent variations, with a maximum 3.8-fold difference previously reported^36,41^. Several factors can influence the FC value, such as age^42,43^, obesity and diet^43^, physical inactivity^44^, and mucus or blood in the fecal sample^45^, though calprotectin itself remains stable for up to one week at room temperature^22,46–48^. Since the same fecal samples were examined with all of the assay kits in this study, those factors do not require consideration. Possible reasons for the variations noted include (1) different assay (method) principles, (2) different antibody for capture or detection, and/or (3) potentially different fecal supernatant extraction efficacy^35,39^. The Buhlmann-E and fCAL-turbo kits use the same antibody, and the strongest correlation was noted between them. Thus, even with different methods and extraction kits, the antibody may be an important factor for result consistency, as the various antibodies used in the different assays would be directed against different complexes of the FC protein.

A variety of cut-off levels for endoscopic remission detection have been reported^35^, which was also observed in the present study. FC cut-off values in UC patients range from 58 to 490 µg/g and in CD patients from 71 to 918 µg/g, though consensus has yet to be established^35^. The present findings suggest that the cut-off is correlated with the kit-dependent actual value for calprotectin, with the cut-off highest for PhiCal and followed in order by fCAL-turbo, Buhlmann-E, and EliA (Table 5), while the median level of actual calprotectin value was also highest for PhiCal, followed by fCAL-turbo, Buhlmann-E, and EliA (Fig. 2). To determine a more reliable cut-off level, investigators will need to consider kit-dependent variations as well. Clinicians are advised to use the same kit for monitoring of IBD activity in individual patients.

The present study has some limitations. First, other assays presently available were not included in the analyses, such as immuno-chromatographic^49,50^, colloidal gold aggregation^51^, and home-based^52,53^ assays, because of their lower frequency of use and level of accuracy. Furthermore, endoscopic score for IBD activity assessment was used, as that is the current gold standard for monitoring endoscopic remission in IBD patients^31–33^, though it would be better to add histological assessment. These limitations should be taken into account when interpreting the results regarding the clinical efficacy of FC for IBD endoscopic scores.

In conclusion, fCAL-turbo, a new rapid fully automated FC kit based on a particle-enhanced turbidimetric immunoassay method, showed strong and significant correlations with other established standard FC kits. Furthermore, moderate correlation with endoscopic activity in UC patients was noted, equivalent to that seen with the other kits. In particular, fCAL-turbo showed excellent performance for predicting endoscopic remission in UC patients, the same as the other standard assays. Based on its advantages including rapid results (15 vs. 150 min) and cost performance (random access testing vs. batch analysis) (Table S1)^29^, use of the fCAL-turbo kit can significantly augment the clinical efficacy of a treat-to-target strategy for IBD cases.

## Materials and methods

### Population

Patient enrollment was performed from October 2016 to November 2018 at Shimane University Hospital and Matsue Seikyo General Hospital in Japan as part of our previous prospective studies^17,18^. For the present investigation, fecal samples obtained in the previous studies were used. The maximum frozen storage period was three years and there were no more than two freeze–thaw cycles. Although calprotectin protein is known to remain stable for several days even at room temperature and after freeze–thaw cycles^47,48^, the quality of the fecal samples was verified, and there were no significant differences for representative FC values between those previous studies and the present. Information for the enrolled patients was analyzed in a retrospective manner. Enrolled patients, as in our previous studies^17,18^, were individuals with a previously established diagnosis of UC or CD, and scheduled to undergo a colonoscopy or balloon-assisted endoscopy (BAE). Excluded were those with colon cancer, acute or chronic gastrointestinal infection including *Clostridioides difficile* and cytomegalovirus, an artificial anus, regular intake of aspirin and/or other nonsteroidal anti-inflammatory drugs that can induce mucosal injury and might increase FC levels^54^, or unable to provide a fecal sample. As for patient demographics, gender, age, disease activity, duration, location, and behavior, and concomitant medications being taken at the time of fecal collection were noted. Clinical UC or CD disease activity was evaluated on the day of the endoscopy examination by use of a partial Mayo score (pMayo, endoscopic subscore removed, remission: 0–2 points, active stage: 3–9 points)^55^ or CD activity index (CDAI; remission: 0–150 points, active stage: > 150 points)^56^, respectively. Assessment of disease extent, location, and behavior was done using the Montreal classification^57^. The study protocol was approved by the institutional review board of both hospitals (Shimane University review board/Hospital review board) and performed in adherence to the Helsinki Declaration. Each patient provided written informed consent for participation.

### Fecal samples

Fecal samples were collected at the first bowel movement in the morning^22^ within three days before a bowel preparation for a colonoscopy procedure, and brought to the hospital or sent by postal mail immediately after collection. Upon arrival, fecal samples were immediately frozen at − 20 °C without a preservative. The maximum time lag from fecal collection to frozen state was within 24 h, during which period calprotectin proteins in fecal samples have been proven to remain stable at room temperature^22,46–48^.

### Measurement of fecal calprotectin level

FC levels were determined with the same fecal sample using each of the following four kits, according to the protocol of manufacturer. Those included three standard kits; EliA^®^ Calprotectin 2 (Thermo Fisher Scientific, Sweden), PhiCal^®^ Calprotectin ELISA (Immundiagnostik, Germany)^49^, and Buhlmann-E (BÜHLMANN fCAL^®^ ELISA, Bühlmann, Switzerland), and also the new BÜHLMANN fCAL^®^ turbo kit (Bühlmann, Switzerland)^30^. All samples were measured in duplicate under the same pre-analytical conditions, with the mathematical mean used to reflect any imprecision for the patient samples. The characteristics of each kit are shown in Table S1. PhiCal assays were performed by SRL Inc. (Tokyo, Japan) as part of our previous prospective study, while the others included in the present study were performed by Nissui Pharmaceutical Co., Ltd. (Tokyo, Japan), after receiving the preserved fecal samples.

### Endoscopic activity scoring

The UC and CD patients received a polyethylene glycol- or magnesium citrate-based electrolyte solution for bowel preparation prior to the endoscopy. In those with UC, a total colonoscopy was generally performed with a magnifying colonoscope (PCF 260AZI, Olympus, Tokyo, Japan). The findings were evaluated using MES for each of five portions of the colorectum (cecum to ascending colon, transverse, descending, sigmoid colon, rectum). Maximum MES in the colorectum was used as the final endoscopy score for the present study. In all patients with CD, ileocolonoscopy or retrograde BAE using a double-balloon enteroscope EN-450T5 (Fujifilm, Tokyo, Japan) examinations were performed. Endoscopic findings indicating CD were evaluated based on mSES-CD, in which the narrowing score is removed for CD cases. All of the endoscopic procedures were performed by experienced gastroenterologists, with the endoscopic scores independently re-assessed by two expert colonoscopists (K.K., A.O.) who were blinded to the FC results. If there were any discrepancies, the final accepted score was determined based on discussion with the two experts and their supervisor.

### Statistical analysis

Nonparametric data are presented as median and interquartile range or 95% CI. For non-paired non-parametric comparisons, a Mann–Whitney test was performed (two-tailed). For non-paired non-parametric multiple comparisons, a Kruskal–Wallis test with ANOVA was performed (two-tailed), while correlation analyses were performed using Spearman’s rank correlation test (two-tailed). For paired non-parametric multiple comparisons, Dunn’s test with ANOVA was used to analyze differences among nonparametric data for paired samples. ROC curve analysis was utilized to determine AUC and optimal cut-off value for each kit for prediction of endoscopic remission. According to the optimal cut-off value, values for sensitivity, specificity, predictive value, and accuracy were also calculated, along with the 95% CI. Statistical analyses were performed using GraphPad Prism software (version 10.0.3), except for DeLong test and multiple regression analysis results, which were analyzed using the EZR software package (version 1.61, modified version of R commander version 2.8-0).

### Supplementary Information

Supplementary Information.

## Data Availability

The data that support the findings of this study are available from the corresponding author, K.K., upon reasonable request.
